# m6A RNA methylation regulators can contribute to malignant progression and impact the prognosis of bladder cancer

**DOI:** 10.1042/BSR20192892

**Published:** 2019-12-20

**Authors:** Mei Chen, Zhen-yu Nie, Xiao-hong Wen, Yuan-hui Gao, Hui Cao, Shu-fang Zhang

**Affiliations:** Central Laboratory, Affiliated Haikou Hospital of Xiangya Medical College, Central South University, Haikou 570208, China

**Keywords:** bladder cancer, m6A, N6-methyladenosine, prognosis

## Abstract

N6-methyladenosine (m6A) is the most common form of messenger RNA (mRNA) modification. An increasing number of studies have proven that m6A RNA methylation regulators are overexpressed in many cancers and participate in the development of cancer through the dynamic regulation of m6A RNA methylation regulators. However, the prognostic role of m6A RNA methylation regulators in bladder cancer (BC) is poorly understood. In the present study, we downloaded the mRNA expression data from The Cancer Genome Atlas (TCGA) database and the corresponding clinical and prognostic information. The relationship between m6A RNA methylation regulators and clinicopathological variables of BC patients was assessed by the Kolmogorov–Smirnov test. The expression of the m6A RNA methylation regulators was differentially associated with different clinicopathological variables of BC patients. The least absolute shrinkage and selection operator (LASSO) Cox regression model was then applied to identify three m6A RNA methylation regulators. The risk signature was constructed as follows: 0.164FTO − (0.081YTHDC1+0.032WTAP). Based on the risk signature, the risk score of each patient was calculated, and the patients were divided into a high-risk group and a low-risk group. The overall survival (OS) rate of the high-risk group was significantly lower than that of the low-risk group. The risk signature was not only an independent prognostic marker for BC patients but also a predictor of clinicopathological variables. In conclusion, m6A RNA methylation regulators can participate in the malignant progression of BC, and a risk signature with three selected m6A RNA methylation regulators may be a promising prognostic biomarker to guide personalized treatment for BC patients.

## Introduction

Bladder cancer (BC) is the most common malignant tumor of the urinary system. In recent years, both morbidity and mortality have increased [1]. BC is classified as muscle-invasive BC (MIBC) and non-MIBC (NMIBC) according to the degree of tumor invasion. Approximately 30% of patients experience invasion of the muscular layer at the time of diagnosis [2]. The incidence of BC in males is higher than that in females [3]. BC is formed by the interaction of various internal and external factors [4]. So far, the regulatory mechanism of BC occurrence and development has not been fully elucidated.

Recently, reversible N6-methyladenosine (m6A) RNA methylation regulators have been found to provide a new method of post-transcriptional regulation [5]. Geneticists found the methylation of m6A in eukaryotic messenger RNA (mRNA) [6]. RNA methylation modification accounts for more than 60% of all RNA modifications, while m6A RNA methylation is the most common type of RNA methylation modification on higher biological mRNAs. m6A RNA methylation regulators play an important role in regulating mRNA splicing, translation and stability [7]. m6A RNA methylation regulators are widely distributed in various types of RNA, such as mRNA, transport RNA (tRNA) and ribosomal RNA (rRNA) [8]. The dysregulation of m6A regulators leads to decreased cell proliferation, a loss of self-renewal capacity, developmental defects and cell death [9]. m6A RNA methylation regulators are involved in the occurrence and development of many cancers, such as liver cancer [10,11], glioblastoma [12], osteosarcoma [13] and colorectal cancer [14]. The level of modification of transcript m6A is regulated by methyltransferases, binding proteins and demethylases [15]. The methyltransferases, known as the ‘writers’, are responsible for the addition of the methyl group to the nitrogen on the sixth carbon of the aromatic ring of an adenosine residue and mainly include KIAA1429, METTL3, METTL14, RBM15, WTAP and ZC3H13 [16]. The m6A binding proteins, known as the ‘readers’, can preferentially bind the RNA to confer its fate and regulate downstream functions and include HNRNPC, YTHDC1, YTHDC2, YTHDF1 and YTHDF2 [17]. The demethylases, known as the ‘erasers’, can specifically target RNA m6A and mainly include ALKBH5 and FTO [18,19]. METTL3 and METTL14 can form a stable heterodimer core complex that functions in cellular m6A deposition on mammalian nuclear RNAs [20]. METTL14 expression is positively correlated with METTL3 expression and is highly expressed in breast cancer [21]. KIAA1429 is an interacting partner of the methyltransferase complex components [22]. WTAP is a regulatory subunit that is important for the localization of METTL3 and METTL14 into nuclear speckles [23]. FTO is associated with human obesity [24]. FTO is highly expressed in acute myeloid leukemia and can inhibit the differentiation of acute myeloid leukemia cells induced by all-*trans* retinoic acid [25]. ALKBH5 is highly expressed in glioblastoma, and ALKBH5 silencing inhibits the proliferation of glioblastoma [26]. METTL3 is highly expressed in BC and promotes BC proliferation in an m6A-dependent manner [27]. METTL3 can promote BC progression through the AFF4/NF-κB/MYC signaling network [28]. *In vivo* and *in vitro* experiments showed that the METTL3-mA-CDCP1 axis and chemical carcinogens could promote the tumorigenesis of BC [29]. Although an increasing number of studies have proven that m6A RNA methylation regulators play an important role in the occurrence and development of cancer, the relationship between m6A RNA methylation regulators and BC is still not fully clear. Many studies have predicted the prognosis of BC by constructing a prognostic signature based on miRNAs [30] and long noncoding RNAs (lncRNAs) [31], but BC prognosis has not been predicted by constructing a prognostic signature based on m6A RNA methylation regulators.

In the present study, we used data from The Cancer Genome Atlas (TCGA) database to analyze the expression of 13 m6A RNA methylation regulators in BC and their relationship with the clinicopathological features of BC. After Cox univariate analysis and least absolute shrinkage and selection operator (LASSO) Cox regression analysis, we selected three m6A methylation regulators to construct a risk signature. Then, we analyzed the prognostic role of the risk signature in BC. We found that m6A RNA methylation regulators play an important role in the malignant progression of BC and that the risk signature can predict the prognosis of BC patients.

## Materials and methods

### Data sources

RNA-seq transcriptome data and the corresponding clinicopathological and prognostic information for 408 BC patients were obtained from the TCGA database (https://cancergenome.nih.gov/).

### Selection of m6A RNA methylation regulators

There are 13 m6A-related genes in BC and 13 genes are recognized as m6A methylation regulators. Although 13 m6A-related genes have been systematically analyzed in gliomas [32], they have not been systematically analyzed in BC. We obtained mRNA expression data for 13 m6A-related genes from the BC cohort of the TCGA database. We compared the relationship among the expression of 13 m6A-related genes and the clinicopathological variables of patients with BC.

### Bioinformatics analysis

The interaction between m6A RNA methylation regulators was analyzed using the STRING database (http://www.string-db.org/). To determine the prognostic role of m6A RNA methylation regulators in BC patients, we performed Cox univariate analysis using data from the TCGA database. Three m6A RNA methylation regulators were selected for further analysis, and a risk signature was developed using the LASSO Cox regression algorithm [33,34]. The risk score was calculated as follows:

$$\text{Risk}\;\text{score}=\sum_{i=1}^{\text{n}}{\text{Coef}}_{\text{i}}\times x_{\text{i}}$$

where Coefi is the coefficient and x_i_ is the expression value of each selected gene. This formula was used to calculate the risk score for each patient.

### Statistical analysis

All statistical analyses were performed using R software (version 3.5.1). The Wilcoxon’s test was used to compare the expression of m6A RNA methylation regulators between cancer and normal tissues. The relationship between m6A RNA methylation regulators and the clinicopathological characteristics of BC patients was analyzed using the Kolmogorov–Smirnov test. The median risk score was used as a cut-off value to classify patients into a high-risk group and a low-risk group, and the Kaplan–Meier method was used to analyze the difference in overall survival (OS) between the high- and low-risk groups. The chi-square test was used to compare the relationship between the risk score and clinicopathological variables. Cox univariate and multivariate analyses of the relationship between clinicopathological variables and risk score were performed.

## Results

### Expression of m6A RNA methylation regulators in BC

**Figure 1:**
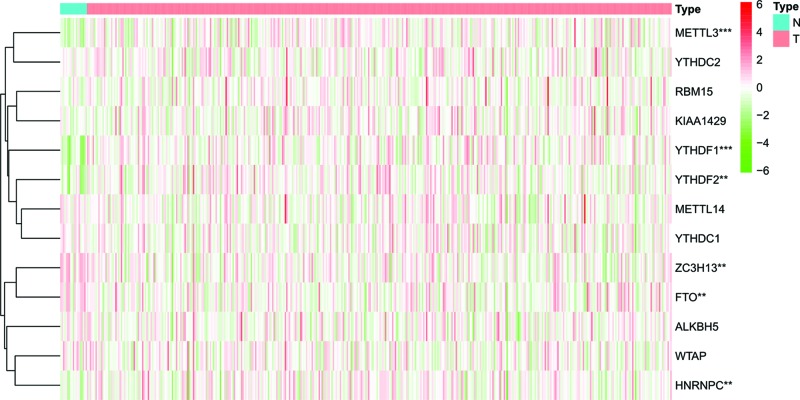
Expression of m6A RNA methylation regulators in BC **P<0.01, ***P<0.001.

We analyzed the expression of 13 m6A RNA methylation regulators in BC using data from the TCGA database. We found that six m6A RNA methylation regulators (METTL3, HNRNPC, YTHDF2, YTHDF1, ZC3H13 and FTO) were differentially expressed in BC tissues compared with normal tissues. Of these regulators, the expression of METTL3 (*P*<0.001), HNRNPC (*P*<0.01), YTHDF2 (*P*<0.01) and YTHDF1 (*P*<0.001) in BC tissues was significantly higher than that in normal tissues, and the expression of ZC3H13 (*P*<0.01) and FTO (*P*<0.01) in normal tissues was significantly higher than that in BC tissues (Figure 1).

### Relationship between the expression of m6A RNA methylation regulators and the clinicopathological features of BC

**Figure 2:**
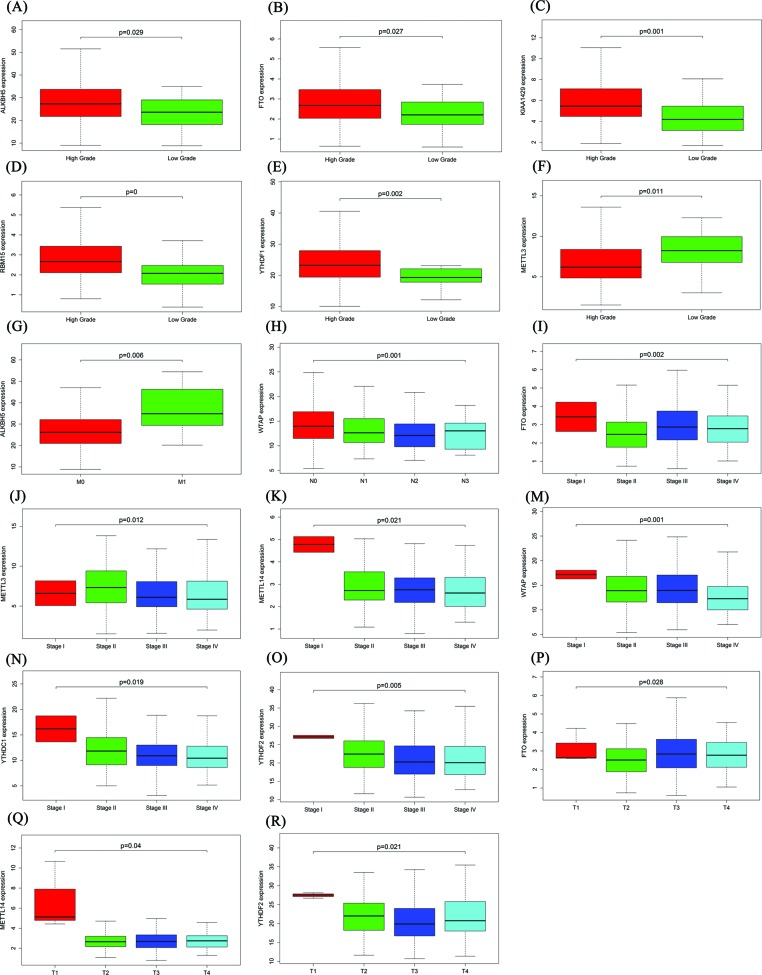
Relationship between m6A RNA methylation regulators and the clinicopathological variables of BC (**A**–**F**) Relationship between m6A RNA methylation regulators and grade. (**G**) Relationship between ALKBH5 and M stage. (**H**) Relationship between WTAP and N stage. (**I**–**O**) Relationship between m6A RNA methylation regulators and stage. (**P**–**R**) Relationship between m6A RNA methylation regulators and T stage.

Because m6A RNA methylation regulators play an important role in tumorigenesis, we further studied the relationship between m6A RNA methylation regulators and BC clinicopathological variables to explore the clinical significance of m6A RNA methylation regulator expression. The results showed that expression of ALKBH5 (*P*=0.029), FTO (*P*=0.027), KIAA1429 (*P*=0.001), RBM15 (*P*=0.000), YTHDF1 (*P*=0.002) and METTL3 (*P*=0.011) was significantly correlated with grade (Figure 2A–F). As the grade increased, the expression of ALKBH5, FTO, KIAA1429, RBM15 and YTHDF1 increased, while the expression of METTL3 decreased. ALKBH5 expression was significantly up-regulated in M1 compared with M0 (*P*=0.006, Figure 2G). The expression of WTAP was significantly correlated with N stage (*P*=0.001, Figure 2H). The expression of FTO (*P*=0.002), METTL3 (*P*=0.012), METTL14 (*P*=0.021), WTAP (*P*=0.001), YTHDC1 (*P*=0.019) and YTHDF2 (*P*=0.005) was significantly correlated with stage (Figure 2I–O). The expression of FTO (*P*=0.028), METTL14 (*P*=0.04) and YTHDF2 (*P*=0.021) was significantly correlated with T stage (Figure 2P–R).

**Figure 3:**
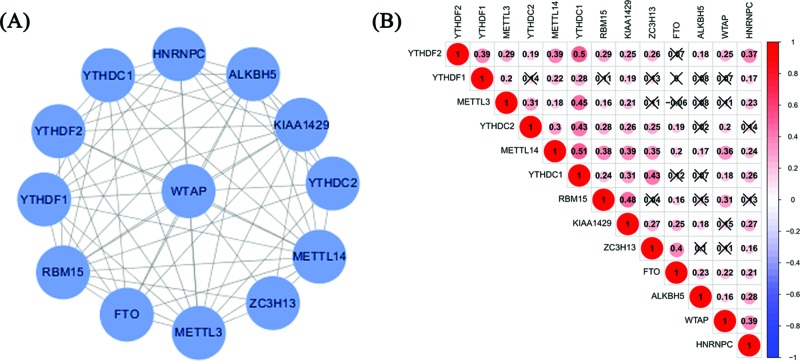
Relationships between m6A RNA methylation regulators (**A**) m6A regulatory gene interactions. (**B**) m6A regulatory gene Spearman correlation analysis.

To further understand the relationship among 13 m6A RNA methylation regulators, we analyzed the interaction and correlation between these genes. In the interaction network, WTAP is at the center and interacts with other m6A RNA methylation regulators (Figure 3A). The correlation between YTHDC1 and METTL14 is the most significant (Figure 3B).

### Prognostic role of m6A RNA methylation regulators in BC

**Figure 4:**
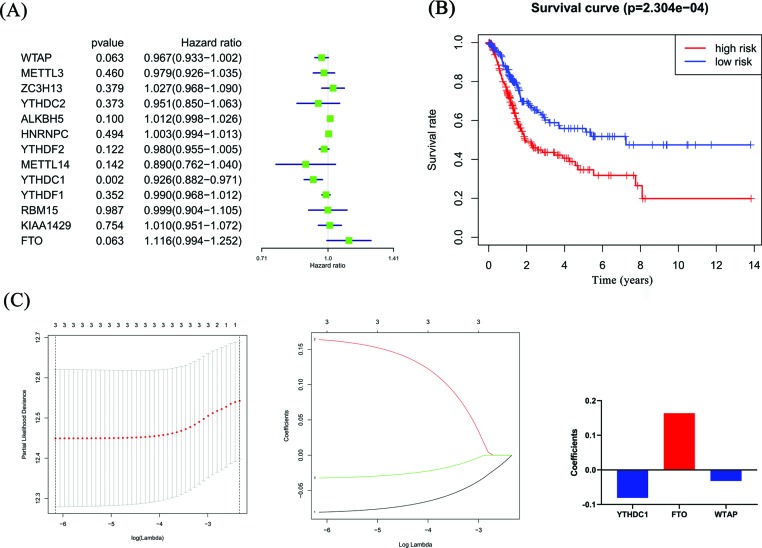
The effect of m6A RNA methylation regulators on the prognosis of BC (**A**) Cox univariate analysis of m6A RNA methylation regulators. (**B**) The relationship between the risk score and the OS of BC patients. (**C**) Coefficients of three m6A RNA methylation regulators.

To investigate the effect of m6A RNA methylation regulators on BC prognosis, we performed Cox univariate analysis (Figure 4A). The risk signature was established by selecting WTAP, YTHDC1 and FTO, and the risk scores of BC patients were calculated through the LASSO Cox regression model, where the coefficient of YTHDC1 was −0.081, the coefficient of FTO was 0.164, and the coefficient of WTAP was −0.032 (Figure 4C). According to the median risk score, patients were divided into low- and a high-risk groups. There was a significant difference in the OS rate between the two groups, and the OS rate of the high-risk group was significantly lower than that of the low-risk group (Figure 4B, *P*=2.304e-04). The 5-year OS rate was 31.8% in the high-risk group and 54.0% in the low-risk group.

### The role of the risk signature in BC patients prognosis

**Figure 5:**
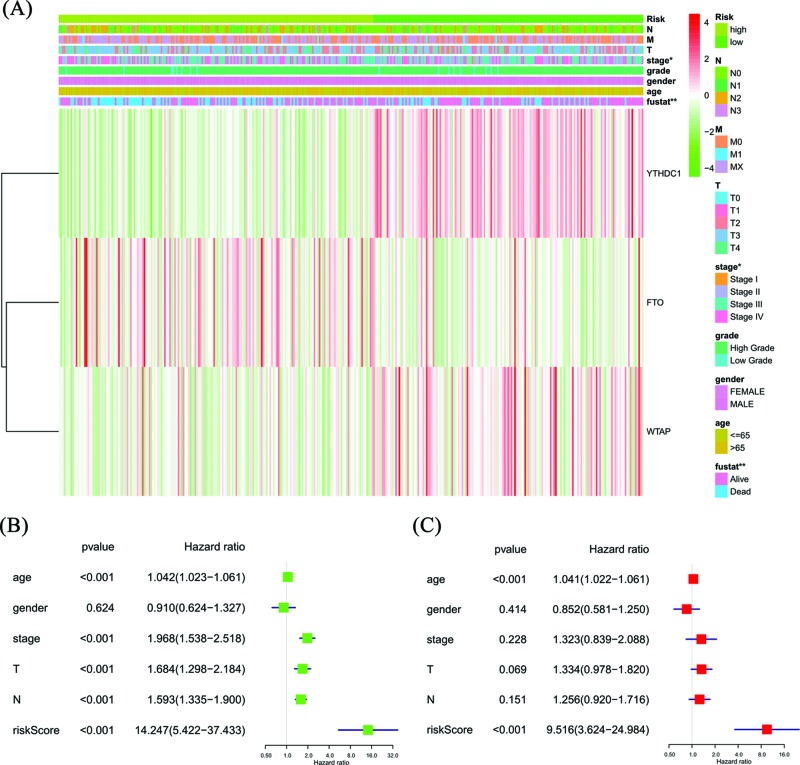
Effects of the risk score and clinicopathological variables on the prognosis of BC patients (**A**) The heat map shows the expression of three m6A RNA methylation regulators and the distribution of clinicopathological variables between the high- and low-risk groups. (**B**) Cox univariate analyses of clinicopathological variables (including the risk score) and overall survival. (**C**) Cox multivariate analyses of clinicopathological variables (including the risk score) and OS.

The heat map shows the expression of the three selected m6A RNA methylation regulators and clinicopathological variables in the high- and low-risk groups. We found significant differences between the two groups in stage (*P*<0.05) and fustat (*P*<0.01) (Figure 5A). We used Cox univariate and multivariate analyses to determine whether the risk signature was an independent predictor. Univariate analyses showed that age, stage, T stage, N stage and risk score were significantly linked with OS (*P*<0.001, Figure 5B). Multivariate analyses showed that age and risk score were significantly linked with OS (*P*<0.001, Figure 5C). These results suggest that the risk signature is a risk factor for BC patients and can independently predict the prognosis of BC patients.

**Figure 6:**
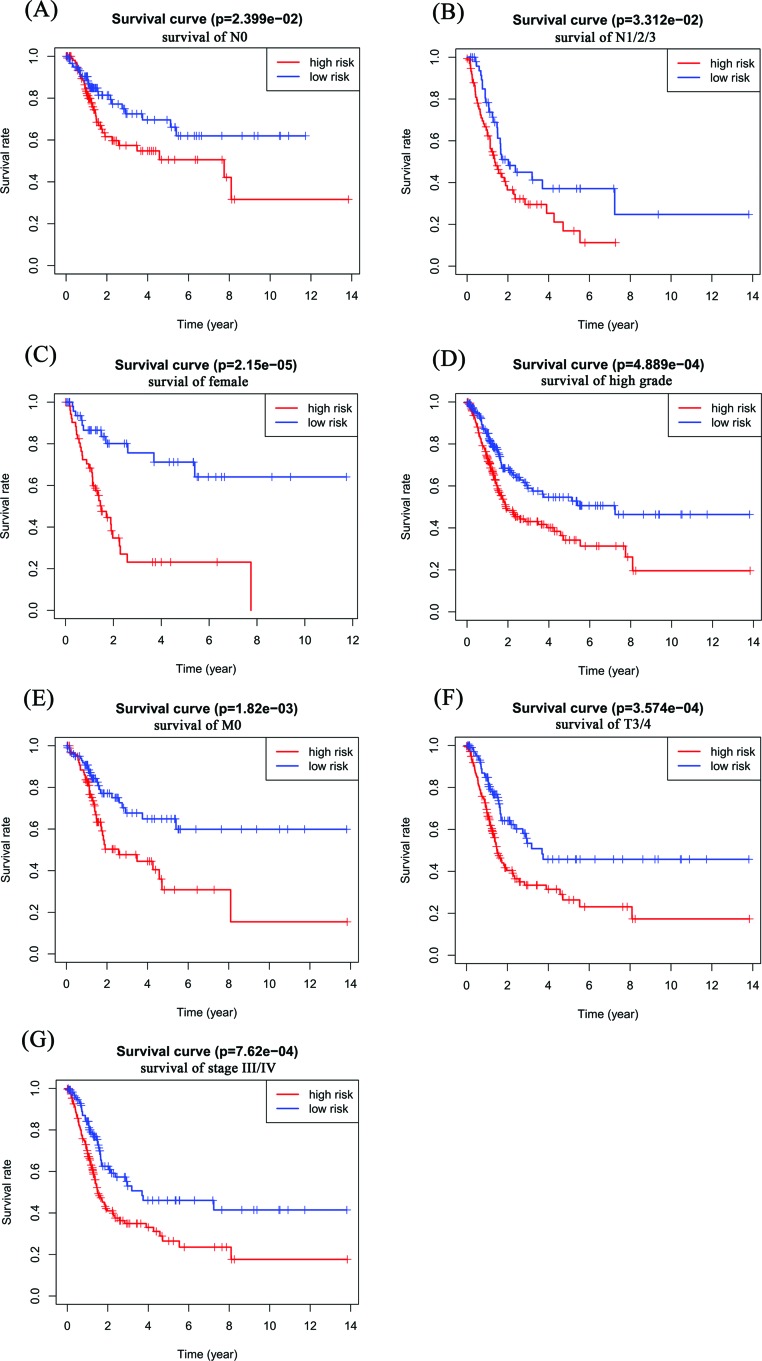
Prognostic value of the risk signature in BC patients classified into specific cohorts Kaplan–Meier survival curve for patients with (**A**) N0 stage, (**B**) N1/2/3 stage, (**C**) female, (**D**) high-grade, (**E**) M0 stage, (**F**) T3/4 stage, and (**G**) stage III/IV.

To detect whether the risk signature adds prognostic value to the clinical system, the patients were also grouped according to clinicopathological variables (gender, grade, M stage, N stage, T stage and stage). The high- and low-risk groups were distinguishable in both the N0 (*P*=2.399e-02, Figure 6A) and N1/2/3 (*P*=3.312e-02, Figure 6B) patient groups, and high-risk patients had a significantly lower OS rate. We found that female patients (*P*=2.15e-05) and those with a high-grade (*P*=4.889e-04), M0 stage (*P*=1.82e-03), T3/4 stage (*P*=3.574e-04) and stage III/IV (*P*=7.62e-04) in the high-risk group had a significantly lower OS rate than those in the low-risk group (Figure 6C–G); however, for male patients and those with a low-grade, M1 stage, T1/2 stage and stage I/II, no significant difference was present (Supplementary Figure S1), which may be due to the small sample size.

## Discussion

BC is one of the most common malignant tumors in the urinary system. The high recurrence rate and drug resistance make the treatment of BC difficult in clinical research. Studies have shown that multiple genes always interact with each other to regulate the development of tumors [35]. m6A RNA methylation regulators play an important role in the development of cancer [36]. Therefore, it is necessary to explore the influence of m6A RNA methylation regulators on BC.

In the present study, we first analyzed the expression of m6A RNA methylation regulators in BC and normal tissues and the relationship between their expression and different clinicopathological variables. The expression of the m6A RNA methylation regulators was differentially associated with different clinicopathological variables of BC patients. METTL3 is an effective therapeutic target for several cancers, such as pancreatic cancer [37], melanoma [38] and lung adenocarcinoma [39]. METTL3 is overexpressed in hepatocarcinoma cells, and its overexpression is associated with a poor prognosis of liver cancer patients [40]. METTL3 is highly expressed in ovarian cancer, and the expression of METTL3 is significantly associated with grade, pT status, pN/pM status and International Federation of Obstetrics and Gynecology (FIGO) stage [41]. All the above studies indicate that METTL3 is a potential oncogene, but in our study, we found that although METTL3 is highly expressed in cancer tissue, it is expressed at low levels in high grade. KIAA1429 is highly expressed in breast cancer and can promote breast cancer proliferation and metastasis *in vivo* and *in vitro* [42]. We found that the expression of KIAA1429 did not differ between BC and normal tissues but was meaningful in grade and highly expressed in high grade. YTHDF1 is overexpressed in colorectal cancer, and YTHDF1 knockdown significantly inhibits Wnt/β-catenin pathway activity in colorectal cancer cells [43]. We found that YTHDF1 is highly expressed in BC and high grade, suggesting a potential carcinogenic effect of YTHDF1 in cancer. ALKBH5 is highly expressed in ovarian cancer. Silencing ALKBH5 can enhance autophagy and inhibit the proliferation and invasion of ovarian cancer cells [44]. We found that ALKBH5 expression did not differ between BC and normal tissues, but the expression of ALKBH5 was positively correlated with grade and M1 stage. These results indicate that ALKBH5 is a potential oncogene that promotes tumor progression. HNRNPC knockdown inhibits the proliferation of breast cancer cells [45]. In our study, HNRNPC was highly expressed in BC. FTO is highly expressed and linked with a poor prognosis in breast cancer [46], but we found that FTO is expressed at low levels in BC. The carcinogenic effects of m6A regulatory genes appear to be controversial. This may be due to the heterogeneity of cancer, which results in the differential expression of m6A regulatory genes in different cancers. The same gene has different roles in different cancers.

Whether m6A RNA methylation regulators have prognostic value in cancer is of great significance [47]. After Cox univariate analysis and LASSO Cox regression analysis, we selected three m6A RNA methylation regulators to construct a risk signature that stratified BC patients into a low-risk group and a high-risk group. Univariate analyses showed that age, stage, T stage, N stage and risk score were significantly associated with OS. Multivariate analyses showed that age and risk score were significantly linked with OS. When patients were also grouped according to clinicopathological variables, the risk signature could also distinguish the OS outcomes of patients with N0 stage, patients with N1/2/3 stage, females, patients with high-grade, patients with M0 stage, patients with T3/4 stage and patients with stage III/IV. The study also has some limitations. We only use the data of TCGA database to verify Cox regression model, and we should also verify it in other public databases.

## Conclusions

m6A RNA methylation regulators are closely related to malignant clinicopathological features, and a risk signature with three selected m6A RNA methylation regulators can independently predict the prognosis of BC patients. Our study provides important evidence for further study of m6A RNA methylation regulators in BC.

## Supplementary Material

Supplementary Figure S1

## Abbreviations

BC: bladder cancer
HNRNPC: Heterogeneous nuclear ribonucleoprotein C
LASSO: least absolute shrinkage and selection operator
mRNA: messenger RNA
m6A: N6-methyladenosine
TCGA: The Cancer Genome Atlas

## References

[1] Rose T.L. and Milowsky M.I. (2016) Improving systemic chemotherapy for bladder cancer. Curr. Oncol. Rep. 18, 27 10.1007/s11912-016-0512-2 26984414

[2] Chang S.S., Bochner B.H., Chou R., Dreicer R., Kamat A.M., Lerner S.P. et al. (2017) Treatment of non-metastatic muscle-invasive bladder cancer: AUA/ASCO/ASTRO/SUO guideline. J. Urol. 198, 552–559 10.1016/j.juro.2017.04.086 28456635 PMC5626446

[3] Abida W., Bajorin D.F. and Rosenberg J.E. (2015) First-line treatment and prognostic factors of metastatic bladder cancer for platinum-eligible patients. Hematol. Oncol. Clin. North Am. 29, 319–328, 10.1016/j.hoc.2014.10.005 25836937

[4] Burger M., Catto J.W., Dalbagni G., Grossman H.B., Herr H., Karakiewicz P. et al. (2013) Epidemiology and risk factors of urothelial bladder cancer. Eur. Urol. 63, 234–241 10.1016/j.eururo.2012.07.033 22877502

[5] Zhao B.S., Roundtree I.A. and He C. (2017) Post-transcriptional gene regulation by mRNA modifications. Nat. Rev. Mol. Cell Biol. 18, 31–42 10.1038/nrm.2016.132 27808276 PMC5167638

[6] Dubin D.T. and Taylor R.H. (1975) The methylation state of poly A-containing messenger RNA from cultured hamster cells. Nucleic Acids Res. 2, 1653–1668 10.1093/nar/2.10.1653 1187339 PMC343535

[7] Yang Z., Li J., Feng G., Gao S., Wang Y., Zhang S. et al. (2017) MicroRNA-145 modulates N(6)-methyladenosine levels by targeting the 3′-untranslated mRNA Region of the N(6)-methyladenosine binding YTH domain family 2 protein. J. Biol. Chem. 292, 3614–3623 10.1074/jbc.M116.749689 28104805 PMC5339747

[8] Visvanathan A. and Somasundaram K. (2018) mRNA traffic control reviewed: N6-Methyladenosine (m(6) A) takes the driver’s seat. Bioessays 40, 1700093 10.1002/bies.201700093 29205437

[9] Liu N. and Pan T. (2016) N6-methyladenosine-encoded epitranscriptomics. Nat. Struct. Mol. Biol. 23, 98–102 10.1038/nsmb.3162 26840897

[10] Zhao X., Chen Y., Mao Q., Jiang X., Jiang W., Chen J. et al. (2018) Overexpression of YTHDF1 is associated with poor prognosis in patients with hepatocellular carcinoma. Cancer Biomark. 21, 859–868 10.3233/CBM-170791 29439311 PMC13078334

[11] Cheng X., Li M., Rao X., Zhang W., Li X., Wang L. et al. (2019) KIAA1429 regulates the migration and invasion of hepatocellular carcinoma by altering m6A modification of ID2 mRNA. Onco Targets Ther. 12, 3421–3428 10.2147/OTT.S180954 31118692 PMC6510231

[12] Cui Q., Shi H., Ye P., Li L., Qu Q., Sun G. et al. (2017) m(6)A RNA methylation regulates the self-renewal and tumorigenesis of glioblastoma stem cells. Cell Rep. 18, 2622–2634 10.1016/j.celrep.2017.02.059 28297667 PMC5479356

[13] Miao W., Chen J., Jia L., Ma J. and Song D. (2019) The m6A methyltransferase METTL3 promotes osteosarcoma progression by regulating the m6A level of LEF1. Biochem. Biophys. Res. Commun. 516, 719–725 10.1016/j.bbrc.2019.06.128 31253399

[14] Li T., Hu P.S., Zuo Z., Lin J.F., Li X., Wu Q.N. et al. (2019) METTL3 facilitates tumor progression via an m(6)A-IGF2BP2-dependent mechanism in colorectal carcinoma. Mol. Cancer 18, 112 10.1186/s12943-019-1038-7 31230592 PMC6589893

[15] Yang Y., Hsu P.J., Chen Y.S. and Yang Y.G. (2018) Dynamic transcriptomic m(6)A decoration: writers, erasers, readers and functions in RNA metabolism. Cell Res. 28, 616–624 10.1038/s41422-018-0040-8 29789545 PMC5993786

[16] Tuncel G. and Kalkan R. (2019) Importance of m N(6)-methyladenosine (m(6)A) RNA modification in cancer. Med. Oncol. 36, 36 10.1007/s12032-019-1260-6 30879160

[17] Zhang C., Fu J. and Zhou Y. (2019) A review in research progress concerning m6A methylation and immunoregulation. Front. Immunol. 10, 922 10.3389/fimmu.2019.00922 31080453 PMC6497756

[18] Li A., Chen Y.S., Ping X.L., Yang X., Xiao W., Yang Y. et al. (2017) Cytoplasmic m(6)A reader YTHDF3 promotes mRNA translation. Cell Res. 27, 444–447 10.1038/cr.2017.10 28106076 PMC5339832

[19] Song Y., Xu Q., Wei Z., Zhen D., Su J., Chen K. et al. (2019) Predict epitranscriptome targets and regulatory functions of N (6)-Methyladenosine (m(6)A) writers and erasers. Evol. Bioinform. Online 15, 1176934319871290 10.1177/1176934319871290 31523126 PMC6728658

[20] Liu J., Yue Y., Han D., Wang X., Fu Y., Zhang L. et al. (2014) A METTL3-METTL14 complex mediates mammalian nuclear RNA N6-adenosine methylation. Nat. Chem. Biol. 10, 93–95 10.1038/nchembio.1432 24316715 PMC3911877

[21] Wu L., Wu D., Ning J., Liu W. and Zhang D. (2019) Changes of N6-methyladenosine modulators promote breast cancer progression. BMC Cancer 19, 326 10.1186/s12885-019-5538-z 30953473 PMC6451293

[22] Schwartz S., Mumbach M.R., Jovanovic M., Wang T., Maciag K., Bushkin G.G. et al. (2014) Perturbation of m6A writers reveals two distinct classes of mRNA methylation at internal and 5′ sites. Cell Rep. 8, 284–296 10.1016/j.celrep.2014.05.048 24981863 PMC4142486

[23] Ping X.L., Sun B.F., Wang L., Xiao W., Yang X., Wang W.J. et al. (2014) Mammalian WTAP is a regulatory subunit of the RNA N6-methyladenosine methyltransferase. Cell Res. 24, 177–189 10.1038/cr.2014.3 24407421 PMC3915904

[24] Dina C., Meyre D., Gallina S., Durand E., Korner A., Jacobson P. et al. (2007) Variation in FTO contributes to childhood obesity and severe adult obesity. Nat. Genet. 39, 724–726 10.1038/ng2048 17496892

[25] Li Z., Weng H., Se R., Weng X., Zuo Z., Li C. et al. (2017) FTO plays an oncogenic role in acute myeloid leukemia as a N-Methyladenosine RNA demethylase. Cancer Cell 31, 127–141 10.1016/j.cell.2016.11.017 28017614 PMC5234852

[26] Zhang S., Zhao B.S., Zhou A., Lin K., Zheng S., Lu Z. et al. (2017) m^6^A demethylase ALKBH5 maintains tumorigenicity of glioblastoma stem-like cells by sustaining FOXM1 expression and cell proliferation program. Cancer Cell 31, 591–606 10.1016/j.ccell.2017.02.013 28344040 PMC5427719

[27] Han J., Wang J.Z., Yang X., Yu H., Zhou R., Lu H.C. et al. (2019) METTL3 promote tumor proliferation of bladder cancer by accelerating pri-miR221/222 maturation in m6A-dependent manner. Mol. Cancer 18, 110 10.1186/s12943-019-1036-9 31228940 PMC6588935

[28] Cheng M., Sheng L., Goa Q., Xiong Q., Zhang H., Wu M. et al. (2019) The m^6^A methyltransferase METTL3 promotes bladder cancer progression via AFF4/NF-κB/MYC signaling network. Oncogene 38, 3667–3680 10.1038/s41388-019-0638-z 30659266

[29] Yang F., Jin H., Que B., Chao Y., Zhang H., Ying X. et al. (2019) Dynamic m(6)A mRNA methylation reveals the role of METTL3-m(6)A-CDCP1 signaling axis in chemical carcinogenesis. Oncogene 38, 4755–4772 10.1038/s41388-019-0755-0 30796352 PMC6756049

[30] Yin X.H., Jin Y.H., Cao Y., Wong Y., Weng H., Sun C. et al. (2019) Development of a 21-miRNA signature associated with the prognosis of patients with bladder cancer. Front. Oncol. 9, 729 10.3389/fonc.2019.00729 31448232 PMC6692470

[31] Lian P., Wang Q., Zhao Y., Chen C., Sun X., Li H. et al. (2019) An eight-long non-coding RNA signature as a candidate prognostic biomarker for bladder cancer. Aging 11, 6930–6940 31479417 10.18632/aging.102225 PMC6756879

[32] Chai R.C., Wu F., Wang Q.X., Zhang S., Zhang K.N., Liu Y.Q. et al. (2019) m(6)A RNA methylation regulators contribute to malignant progression and have clinical prognostic impact in gliomas. Aging 11, 1204–1225 10.18632/aging.101829 30810537 PMC6402513

[33] Sauerbrei W., Royston P. and Binder H. (2007) Selection of important variables and determination of functional form for continuous predictors in multivariable model building. Stat. Med. 26, 5512–5528 10.1002/sim.3148 18058845

[34] Bovelstad H.M., Nygard S., Storvold H.L., Aldrin M., Borgan O., Frigessi A. et al. (2007) Predicting survival from microarray data–a comparative study. Bioinformatics 23, 2080–2087 10.1093/bioinformatics/btm305 17553857

[35] Yu W., Zhao S., Wang Y., Zhao B.N., Zhao W. and Zhou X. (2017) Identification of cancer prognosis-associated functional modules using differential co-expression networks. Oncotarget 8, 112928–112941 10.18632/oncotarget.22878 29348878 PMC5762563

[36] Wang S., Sun C., Li J., Zhang E., Ma Z., Xu W. et al. (2017) Roles of RNA methylation by means of N(6)-methyladenosine (m(6)A) in human cancers. Cancer Lett. 408, 112–120 10.1016/j.canlet.2017.08.030 28867248

[37] Taketo K., Konno M., Asai A., Koseki J., Toratani M., Satoh T. et al. (2018) The epitranscriptome m6A writer METTL3 promotes chemo- and radioresistance in pancreatic cancer cells. Int. J. Oncol. 52, 621–629 29345285 10.3892/ijo.2017.4219

[38] Dahal U., Le K. and Gupta M. (2019) RNA m6A methyltransferase METTL3 regulates invasiveness of melanoma cells by matrix metallopeptidase 2. Melanoma Res. 29, 382–389 10.1097/CMR.0000000000000580 30762711

[39] Lin S., Choe J., Du P., Triboulet R. and Gregory R.I. (2016) The m(6)A methyltransferase METTL3 promotes translation in human cancer cells. Mol. Cell 62, 335–345 10.1016/j.molcel.2016.03.021 27117702 PMC4860043

[40] Chen M., Wei L., Law C.T., Tsang F.H., Shen J., Cheng C.L. et al. (2018) RNA N6-methyladenosine methyltransferase-like 3 promotes liver cancer progression through YTHDF2-dependent posttranscriptional silencing of SOCS2. Hepatology 67, 2254–2270 10.1002/hep.29683 29171881

[41] Hua W., Zhao Y., Jin X., Yu D., He J., Xie D. et al. (2018) METTL3 promotes ovarian carcinoma growth and invasion through the regulation of AXL translation and epithelial to mesenchymal transition. Gynecol. Oncol. 151, 356–365 10.1016/j.ygyno.2018.09.015 30249526

[42] Qian J.Y., Gao J., Sun X., Cao M.D., Shi L., Xia T.S. et al. (2019) KIAA1429 acts as an oncogenic factor in breast cancer by regulating CDK1 in an N6-methyladenosine-independent manner. Oncogene 38, 6123–6141 10.1038/s41388-019-0861-z 31285549

[43] Bai Y., Yang C., Wu R., Huang L., Song S., Li W. et al. (2019) YTHDF1 regulates tumorigenicity and cancer stem cell-like activity in human colorectal carcinoma. Front. Oncol. 9, 332 10.3389/fonc.2019.00332 31131257 PMC6509179

[44] Zhu H., Gan X., Jiang X., Diao S., Wu H. and Hu J. (2019) ALKBH5 inhibited autophagy of epithelial ovarian cancer through miR-7 and BCL-2. J. Exp. Clin. Cancer Res. 38, 163 10.1186/s13046-019-1159-2 30987661 PMC6463658

[45] Wu Y., Zhao W., Liu Y., Tan X., Li X., Zou Q. et al. (2018) Function of HNRNPC in breast cancer cells by controlling the dsRNA-induced interferon response. EMBO J. 37, e99017 10.15252/embj.201899017 30158112 PMC6276880

[46] Niu Y., Lin Z., Wan A., Chen H., Liang H., Sun L. et al. (2019) RNA N6-methyladenosine demethylase FTO promotes breast tumor progression through inhibiting BNIP3. Mol. Cancer 18, 46 10.1186/s12943-019-1004-4 30922314 PMC6437932

[47] Pan Y., Ma P., Liu Y., Li W. and Shu Y. (2018) Multiple functions of m(6)A RNA methylation in cancer. J. Hematol. Oncol. 11, 48 10.1186/s13045-018-0590-8 29587823 PMC5870302

