# Supplementary material for: m6A RNA methylation regulators can contribute to malignant progression and impact the prognosis of bladder cancer
Source: Biosci Rep. 2019 Dec 20;39(12):BSR20192892. doi: 10.1042/BSR20192892 (PMC6923333; doi:10.1042/BSR20192892)
Supplement: Supplementary Figure S1 [file BSR-2019-2892_supp.pdf]

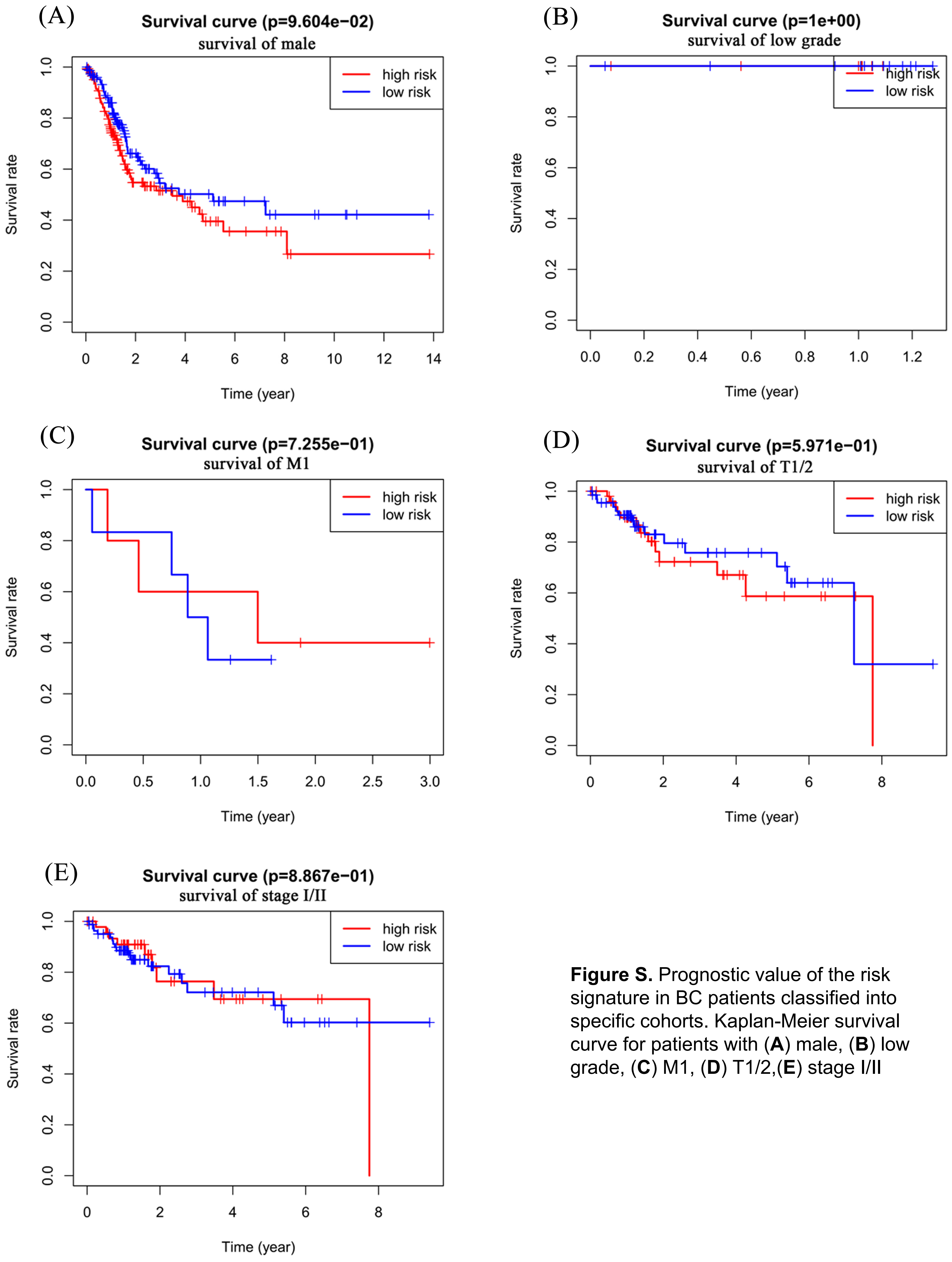

Figure S. Prognostic value of the risk signature in BC patients classified into specific cohorts. Kaplan-Meier survival curve for patients with (**A**) male, (**B**) low grade, (**C**) M1, (**D**) T1/2,(**E**) stage I/II
